# Molecular Characterization of *Candida africana* in Genital Specimens in Shanghai, China

**DOI:** 10.1155/2015/185387

**Published:** 2015-11-19

**Authors:** Yang Hu, Aihua Yu, Xiangming Chen, Guojiang Wang, Xiaobo Feng

**Affiliations:** ^1^Department of Dermatology, Shanghai Jiading Nanxiang Hospital, Shanghai 201802, China; ^2^Medical Mycology Laboratory, Department of Dermatology, Xinhua Hospital, Shanghai Jiao Tong University School of Medicine, Shanghai 200092, China

## Abstract

*Candida africana*, an emerging yeast pathogen, is closely related to *Candida albicans* and most commonly involved in vulvovaginal candidiasis (VVC). However, its prevalence in candidal balanoposthitis is still unclear. In this study, the prevalence of *C. africana* in both candidal balanoposthitis and VVC in a sexually transmitted diseases (STD) clinic in Shanghai, China, was analyzed, and the molecular characterization and susceptible profiles of *C. africana* isolates were investigated. As results, *C. africana* was only isolated in 5 out of 79 (6.3%) cases of candidal balanoposthitis rather than cases with vulvovaginal candidiasis. Among them, 4 out of 5 isolates share the same genotype of DST 782 with an isolate from vaginal swab in Japan published previously. All *C. africana* isolates were susceptible to amphotericin B, flucytosine, fluconazole, itraconazole, voriconazole, posaconazole, caspofungin, and micafungin.

## 1. Introduction

Emerging* Candida* species have been detected in cases of candidiasis by using molecular identification, and these species actually belong to diverse species complexes [1–15].* Candida africana* is an emerging pathogen that was proposed as a new species or variety within the* Candida albicans* complex since 2001 [16–20]. Due to phenotypic resemblance and unavailability of commercial tools, this pathogen was readily misidentified as* C. albicans* in clinical laboratory. Although* C. africana* is also germ tube-positive, it exhibits differences in producing chlamydospores, morphology on CHROMagar* Candida* medium, and assimilating results with* C. albicans* [16, 21]. However, precise identification usually relies on molecular techniques such as HWP1 gene amplification or pyrosequencing of a short fragment of the internal transcribed spacer region 2 (ITS2) [1, 22, 23]. Previous studies showed that* C. africana* differed from* C. albicans* in clinical distribution, and the former was most commonly involved in vulvovaginal candidiasis (VVC), implicating its tropism for vagina [23, 24]. When only the* C. albicans* complex isolates from vaginal samples were considered,* C. africana* was much more prevalent than* C. dubliniensis* [23]. Furthermore, differences in pathogenicity, adherence ability, and biofilm formation were observed between* C. africana* and* C. albicans* in previous studies [21, 23, 24], implicating the necessity to differentiate them in clinical laboratory.

Vulvovaginal candidosis is one of the most common infection types and accounts for 40%–50% cases of infectious vulvovaginitis. Candidal balanoposthitis, the most frequent genital infection occurring in man, was revealed to be associated with sexual transmission [25, 26]. In two recent studies, similar genotype distributions between* C. albicans* strains causing balanoposthitis and those causing VVC were confirmed, which imply the sexual transmission of genital* C. albicans* infection [25]. Although global distribution of* C. africana* in VVC was found, no information on the prevalence of this emerging pathogen in candidal balanoposthitis was available. This study will reveal the distribution, antifungal profiles, and genetic characterization of* C. africana* isolates from both candidal balanoposthitis and VVC patients in Shanghai, China.

## 2. Materials and Methods

### 2.1. Strains and Conventional Identification

All genital samples were collected from patients presenting with VVC or candidal balanoposthitis in sexually transmitted diseases (STD) clinic of our hospital between December 2013 and December 2014. A total of 166 independent* Candida* isolates (79 from cases of balanoposthitis and 87 from cases of vulvovaginitis) were recovered. All isolates were phenotypically characterized using conventional methods. Germ tube test was performed by inoculating 0.5 mL of fetal calf serum with a loopful of yeast. Germ tubes were produced by* C. albicans* complex within 3 h at 37°C. Chlamydospores production was assessed by culturing yeast on cornmeal agar at 30°C for 5 days. Appearance of the colonies on CHROMagar* Candida* medium and assimilation profiles with the API 20C AUX system (bioMérieux, Marcy l'Etoile, France) were also recorded according to the manufacturer's instructions.

### 2.2. Molecular Identification by Use of HWP1 Gene Amplification

Isolates showing green color colony on the CHROMagar* Candida* medium and germ tube-positive in serum were chosen for molecular analysis. Genomic DNA was extracted from each isolate with the MasterPure Yeast DNA Purification kit (Epicentre Biotechnologies, Madison, WI) according to the manufacturer's instructions. HWP1 gene amplification was performed to distinguish* C. albicans*,* C. dubliniensis*, and* C. africana* on the basis of the distinct size of the amplicons as previously described [22]. Reference strains used as control in this study were* C. albicans* ATCC10231,* C. dubliniensis* CD36, and* C. africana* Caf1.

### 2.3. ITS Sequencing and Multilocus Sequence Typing

All* C. africana* isolates identified by use of HWP1 gene amplification were subjected to ITS sequencing using primers ITS1 and ITS4 as previously depicted [3]. The* C. africana* isolates were further identified based on the ITS2 sequence as described previously [23]. The MLST scheme using seven housekeeping genes, namely, AAT1a, ACC1, ADP1, MPIb, SYA1, VPS13, and ZWF1b, was conducted for the* C. africana* isolates obtained herein as previously described [18, 27]. Sequencing was performed on ABI 3730 automatic sequencer (Applied Biosystems, Foster City, CA). The seven loci were sequenced in both directions, and sequence data were checked manually for positions of homozygotic or heterozygotic polymorphisms. Single nucleotide polymorphisms that occurred in sequence were included in analysis. Allele numbers for each locus were assigned on the basis of comparison of sequences obtained here to those deposited at the public MLST database (http://pubmlst.org/calbicans/). The composite profile of the seven allele numbers for an isolate determined the isolate's diploid sequence type (DST). A unique DST will be assigned if our isolates differ from isolates published in one or more nucleotides at any of the seven loci.

### 2.4. *In Vitro* Antifungal Susceptibility Testing

All* C. africana* isolates obtained herein were tested for* in vitro* susceptibility to amphotericin B, flucytosine, fluconazole, itraconazole, voriconazole, and posaconazole (Sigma, St. Louis, MO, USA), caspofungin (Merck & Co., Inc.), and micafungin (Astellas Pharma) by using broth microdilution method as described in CLSI document M27-A3 and M27-S4. The MIC values were read following 24 h of incubation and determined visually as the lowest concentration in which prominent decrease in turbidity was observed excepting for amphotericin B which is defined as the lowest concentration in which the absence of turbidity is observed. The recently revised CLSI clinical breakpoints (CBPs) values for* C. albicans* were used as reference [28]. Quality control was performed as recommended in CLSI documents using* C. parapsilosis* ATCC22019 and* C. krusei* ATCC 6258.

## 3. Results

Among the 166* Candida* isolates, 129 (77.7%) isolates were germ tube-positive and showed green color on CHROMagar* Candida* medium. However, 5 out of 129 (3.9%) isolates produced smaller and deeper turquoise-green colonies compared with the other isolates. These five isolates failed to produce chlamydospores on rice tween 80's agar after 5 days of incubation at 28°C. The five unique strains were all isolated from glans penis or foreskin swab samples, and none was isolated from vaginal samples.

**Figure 1:**
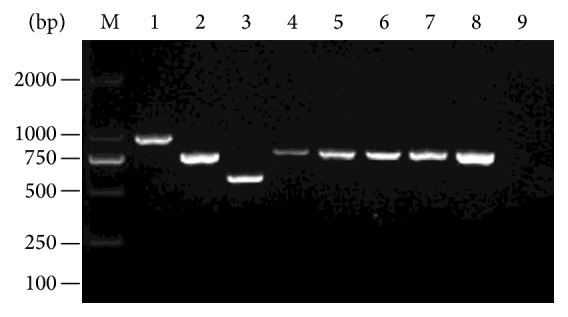
Agarose gel electrophoresis of PCR products. Species or variety is depicted in brackets. Lanes: M, DL2000 ladder. 1, ATCC10231 (*C. albicans*). 2, Caf1 (*C. africana*). 3, CD36 (*C. dubliniensis*). 4, XH455 (*C. africana*). 5, XH824 (*C. africana*). 6, XH520 (*C. africana*). 7, XH644 (*C. africana*). 8, XH874 (*C. africana*). 9, negative control.

Among the 129 isolates presumptively identified as* C. albicans*, 124 (96.1%) were found to be* C. albicans* isolates and 5 (3.9%) were found to be* C. africana* isolates on the basis of HWP1 gene amplification. No* C. dubliniensis* isolates were found in this study. All five* C. africana* isolates yielded an amplicon of size ~750 bp identical to that of the* C. africana* reference strain Caf1 (Figure 1). All five* C. africana* isolates shared the identical ITS sequence which differ from* C. albicans* in one position located within the 35 bp ITS2 signature sequence. The result of the molecular identification was consistent with the phenotypic analysis.

DNA sequencing of the fragments from the coding region of each of the seven genes resulted in a total of 2,883 nucleotides for each* C. africana* isolate. Among them, 4* C. africana* isolates shared the genotype DST 782 of* C. africana* strain JIMS500002 assigned by MLST database at the seven loci, AAT1a, ACC1, ADP1, MPIb, SYA1, VPS13, and ZWF1b. The remaining 1 isolate shared a common genotype DST 182. All the allele numbers of seven loci and their corresponding DSTs for 5 Shanghai* C. africana* isolates and reference* C. africana* strains were summarized in Table 1.

All 5* C. africana* isolates were susceptible to fluconazole (MIC range, 0.12–0.25 *μ*g/mL), itraconazole (MIC range, 0.03–0.12 *μ*g/mL), voriconazole (MIC range, <0.016–0.016 *μ*g/mL), caspofungin (MIC range, 0.06–0.25 *μ*g/mL), and micafungin (MIC range, <0.03–0.06 *μ*g/mL). These isolates also exhibited low MICs to amphotericin B (MIC range, 0.03–0.12 *μ*g/mL), flucytosine (MIC range, <0.12–0.12 *μ*g/mL), and posaconazole (0.016–0.03) (see Table 2).

## 4. Discussion

Simple PCR assay was applied for the identification of cryptic species within* Candida* spp. [2, 3, 9, 22]. Here, the result of the HWP1 gene amplification was consistent with the sequencing of the internal transcribed spacer region 2 (ITS2) for identification of* C. africana*. Additionally,* C. africana* could also be distinguished from* C. albicans* on the basis of its appearance on CHROMagar, with the former producing smaller and deeper turquoise-green colonies than* C. albicans*, which may be used as a preliminary screening method. No* C. africana* isolated herein produce chlamydospores, which is consistent with previous reports [16, 21, 27].

Although* C. africana* has been isolated from vaginal specimens worldwide [17], no* C. africana* was identified in cases with VVC in our study, which was similar to another two studies in which* C. africana* was not identified among 195 and 98 vaginal* C. albicans* complex isolates from Turkey and Malaysia, respectively [29, 30]. We think that this situation may result from regional differences or limited clinical samples. The present study revealed that* C. africana* consists of 6.3% of isolates from cases of candidal balanoposthitis. The mean age of the five male patients with candidal balanoposthitis due to* C. africana* was 31.6 years (range 24–42 years). To our knowledge, this is the first time to study the prevalence of this emerging pathogen in candidal balanoposthitis.

We conducted a well-established MLST scheme to determine the genetic relatedness of* C. africana* isolates based on seven housekeeping genes. However, no* C. africana* was isolated from vaginal samples which hampered analyzing genotype distributions between* C. africana* strains causing balanoposthitis and those causing VVC. The seven gene fragments used for MLST of* C. africana* yielded two distinct DSTs among the five* C. africana* strains. Among them, 4 out of 5 isolates share the same genotype of DST 782 with an isolate from vaginal swab in Japan published previously, implicating the possibility of sexual transmission of genital* C. africana* infection. In another report, a white German patient with balanitis and his African girlfriend with vaginal mycoses caused by* C. africana* were seen at the institute of mycology in Berlin [31]. Thus, genotyping of* C. africana* isolates from patient and sexual partner may indicate the sexual transmission of genital* C. africana* infection. The genotype of DST 782 was not found in other countries up to now, and additionally special genotype was identified in a study in India [27]. This means that the distribution of* C. africana* genotype may be based partially on geographical variation. More* C. africana* isolates should be included in the MLST analysis in the future to confirm this hypothesis, because only 24* C. africana* isolates have been typed by the MLST scheme till now. Because only three genotypes (DSTs) were available for* C. africana* at MLST website, more genetic markers and isolates from worldwide should be included in the molecular typing scheme to elucidate the global epidemiology of this emerging pathogen.

In agreement with other reports [23], all* C. africana* isolates tested were found to be susceptible (with very low MICs) to all antifungal agents, which would be appropriate for treating candidal balanoposthitis due to this emerging pathogen.

## Figures and Tables

**Table 1:** Multilocus sequence typing profile of the five Shanghai *C. africana* isolates together with three reference strains with distinct genotypes.

Isolate number	ST	AAT1	ACC1	ADP1	MPIb	SYA1	VPS13	ZWF1b
AM2003/0025	182	33	7	32	**26**	2	61	48
JIMS500002	782	33	7	32	**43**	2	61	48
VPCI84/P/13	2191	33	7	32	**135**	2	61	48
XH455^*∗*^	782	33	7	32	**43**	2	61	48
XH824^*∗*^	782	33	7	32	**43**	2	61	48
XH520^*∗*^	182	33	7	32	**26**	2	61	48
XH644^*∗*^	782	33	7	32	**43**	2	61	48
XH874^*∗*^	782	33	7	32	**43**	2	61	48

^*∗*^Isolates recovered in this study.

**Table 2:** *In vitro* susceptibility of the five Shanghai *C. africana* isolates.

Isolate number	Patient	Age	Minimum inhibitory concentration (*µ*g/mL)							
			AMB	5-FC	FLC	ITC	VOR	POS	CAS	MIC
XH455	M	27	0.12	<0.12	0.12	0.12	0.016	0.016	0.06	0.03
XH824	M	42	0.06	<0.12	0.12	0.06	<0.016	0.016	0.06	<0.03
XH520	M	41	0.12	0.12	0.25	0.03	0.016	0.03	0.12	0.06
XH644	M	24	0.03	0.12	0.12	0.12	0.016	0.016	0.25	<0.03
XH874	M	24	0.12	<0.12	0.12	0.03	<0.016	0.016	0.06	<0.03

AMB, amphotericin B; 5-FC, flucytosine; FLC, fluconazole; ITC, itraconazole; VOR, voriconazole; POS, posaconazole; CAS, caspofungin; MIC, micafungin.
